# Cranberry-derived proanthocyanidins impair virulence and inhibit quorum sensing of *Pseudomonas aeruginosa*

**DOI:** 10.1038/srep30169

**Published:** 2016-08-09

**Authors:** Vimal B. Maisuria, Yossef Lopez-de Los Santos, Nathalie Tufenkji, Eric Déziel

**Affiliations:** 1 Department of Chemical Engineering, McGill University, 3610 University Street, Montreal, Quebec, Canada; 2 INRS-Institut Armand-Frappier, 531 boul. des Prairies, Laval, Québec, Canada

## Abstract

Bacteria have evolved multiple strategies for causing infections that include producing virulence factors, undertaking motility, developing biofilms, and invading host cells. *N*-acylhomoserine lactone (AHL)-mediated quorum sensing (QS) tightly regulates the expression of multiple virulence factors in the opportunistic pathogenic bacterium *Pseudomonas aeruginosa*. Thus, inhibiting QS could lead to health benefits. In this study, we demonstrate an anti-virulence activity of a cranberry extract rich in proanthocyanidins (cerPAC) against *P. aeruginosa* in the model host *Drosophila melanogaster* and show this is mediated by QS interference. cerPAC reduced the production of QS-regulated virulence determinants and protected *D. melanogaster* from fatal infection by *P. aeruginosa* PA14. Quantification of AHL production using liquid chromatography-mass spectrometry confirmed that cerPAC effectively reduced the level of AHLs produced by the bacteria. Furthermore, monitoring QS signaling gene expression revealed that AHL synthases LasI/RhlI and QS transcriptional regulators LasR/RhlR genes were inhibited and antagonized, respectively, by cerPAC. Molecular docking studies suggest that cranberry-derived proanthocyanidin binds to QS transcriptional regulators, mainly interacting with their ligand binding sites. These findings provide insights into the underlying mechanisms of action of a cerPAC to restrict the virulence of *P. aeruginosa* and can have implications in the development of alternative approaches to control infections.

As antibiotic resistance in microbial pathogens embodies a global threat to public health, it demands the development of novel strategies for managing microbial infections. The long-term effectiveness of most antibiotic treatments is restricted by both pathogen drug resistance and non-target effects on the host’s commensal microbial community. Over the last decade, research on antimicrobials has shifted towards an alternative approach to combat pathogens using anti-infective drugs that selectively interrupt virulence pathways to help prevent or cure bacterial infections. Anti-infective drugs that do not perturb survival or viability of bacterial pathogens should be less likely to promote resistance than conventional antibiotics^1^,^2^. Until now, the development of anti-infective synthetic drugs has been limited to the laboratory and preclinical studies^2^,^3^,^4^. Natural bioactive compounds derived from plant species show promising therapeutic properties to combat the emerging resistance in microbial pathogens, which can be exploited as next generation anti-infective drugs.

The fruit of the American cranberry (*Vaccinium macrocarpon* L.) has been anecdotally reported as a natural remedy for urinary tract infections for centuries^5^,^6^. Accordingly, a growing number of studies have examined the potential anti-oxidant^7^, anti-adhesion^8^,^9^,^10^,^11^, anti-motility^12^,^13^,^14^,^15^,^16^,^17^ and anti-cancer^18^,^19^ properties of cranberry-derived compounds. A number of these studies focused on the bioactivity of a specific fraction of cranberry phytochemicals known as proanthocyanidins (cPACs). Research shows that these condensed tannins hinder bacterial attachment to cellular or biomaterial surfaces^8^,^11^,^20^, impair motility of the pathogens *Pseudomonas aeruginosa*, *Escherichia coli* and *Proteus mirabilis*^12^,^13^,^14^,^15^,^16^,^17^, and can induce a state of iron limitation in uropathogenic *E. coli*^21^. O’May *et al.*^15^ also reported that a different cranberry derived material, namely, cranberry powder derived from whole, crushed cranberries, impairs the surface-associated swarming motility of *P. aeruginosa* strain PAO1 while enhancing biofilm formation. Also, cPACs block invasion of intestinal pathogens as a result of rearrangement of host cell cytoskeleton *in vitro*^20^. While many studies have suggested that consumption of cranberry-derived materials, namely cranberry capsules and cranberry juice, is effective in preventing bacterial infections^22^,^23^,^24^,^25^,^26^, few have looked at the effects of these cranberry-derived materials *in vitro* and *in vivo* after consumption^24^,^27^,^28^. Indeed, the effect of bioactive cPACs on bacterial virulence *in vivo* and mechanisms by which they do so are poorly understood. To date, not much attention has been given to the anti-virulence properties of cPACs.

*P. aeruginosa* is an opportunistic and versatile *γ*-proteobacterium that readily develops antibiotic resistance and is responsible for various infections affecting immunocompromised individuals, such as those suffering from cystic fibrosis^29^,^30^,^31^. *P. aeruginosa* regulates most of its virulence factors in a cell density-dependent manner via cell-to-cell communication, commonly known as quorum sensing (QS)^32^. *P. aeruginosa* has two major *N*-acylhomoserine lactone (AHL)-based QS pathways, the Las and Rhl QS systems, and one 2-alkyl-4-(1H)-quinolone (AQ)-based QS system, which function in a cascade manner^33^,^34^. The Las system is positioned at the top of the QS hierarchy and uses *N*-3-(oxo-dodecanoyl)-L-homoserine lactone (3-oxo-C_12_-HSL) as its signal molecule, and involves LasI and LasR as the synthase and regulator, respectively^35^,^36^. The Rhl system uses *N*-butanoyl-L-homoserine lactone (C_4_-HSL) as its signal, and involves RhlI and RhlR as the synthase and regulator, respectively^35^,^36^. The LasR initiates the QS regulatory systems, partially activates the transcription of RhlR and other regulators of *Pseudomonas* quinolone signal (PQS) and integrated quorum sensing (IQS) systems^33^,^37^. The complex QS regulation network influences, both positively and negatively, the transcription of possibly 5–10% genes of *P. aeruginosa*^38^,^39^. The QS system is an essential part of the organism’s virulence and is required to establish infection in the mammalian host^40^.

Bacterial QS is considered an attractive therapeutic target in efforts to diminish bacterial virulence by reducing the level of QS signals, or by interfering with other QS system targets^41^,^42^. Several attempts were made to find suitable candidate molecules with anti-QS and anti-biofilm activity against *P. aeruginosa*^43^. Experimental and modeling studies suggest that evolution and spread of resistance in bacteria against QS inhibiting compounds may be occurring^44^,^45^. Thus, the identification of new compounds aimed at inhibiting bacterial QS will mitigate the emergence of antimicrobial resistance in bacterial pathogens.

The goal of this study was to examine the anti-virulence potential of a cranberry extract rich in proanthocyanidins (cerPAC) in combating the opportunistic human pathogen *P. aeruginosa*. Our results clearly show the anti-infectious properties of a cerPAC against *P. aeruginosa* strain PA14 and reveal the multi-modal action of cerPAC in impairing QS function.

## Results

### A cranberry extract rich in proanthocyanidins inhibits *P. aeruginosa* virulence towards *D. melanogaster*

**Figure 1:**
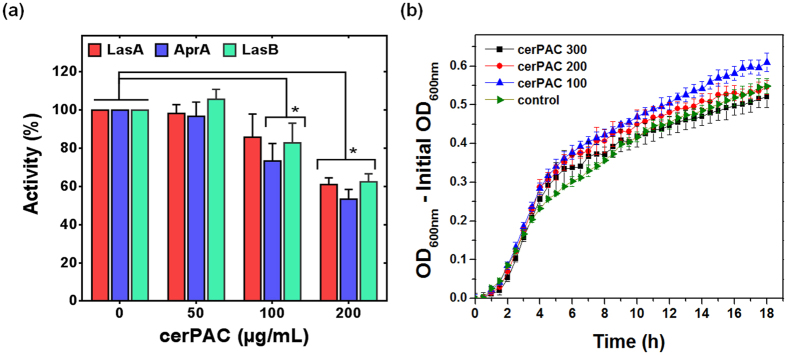
(**a**) Inhibition of virulence determinants and (**b**) growth curves of *P. aeruginosa* PA14 in absence or presence of different cerPAC concentrations. LasA: staphylolytic protease, LasB: elastase and AprA: alkaline protease. Results are expressed as means and standard deviations (SD) of triplicate enzyme assays (*p < 0.001). Bacterial growth (OD_600_) was monitored at 37 °C for 18 h in TSB medium. Error bars with average data points of growth kinetics represent the standard deviation of values obtained from four replicates. Abbreviations: cerPAC x, Cranberry extract rich in proanthocyanidins at x μg mL^−1^ (e.g., cerPAC 300 indicates cerPAC at 300 μg mL^−1^).

Treatment with cerPAC significantly inhibited the staphylolytic (LasA, *F*_*3,8*_ = 21.41, *p* < 0.001), elastolytic (LasB, *F*_*3,8*_ = 84.29, *p* < 0.001) and alkaline proteolytic (AprA, *F*_*3,8*_ = 34.41, *p* < 0.001) activities of *P. aeruginosa* PA14 (Fig. 1a). Importantly, this inhibition was achieved without affecting bacterial growth (Fig. 1b).

**Figure 2:**
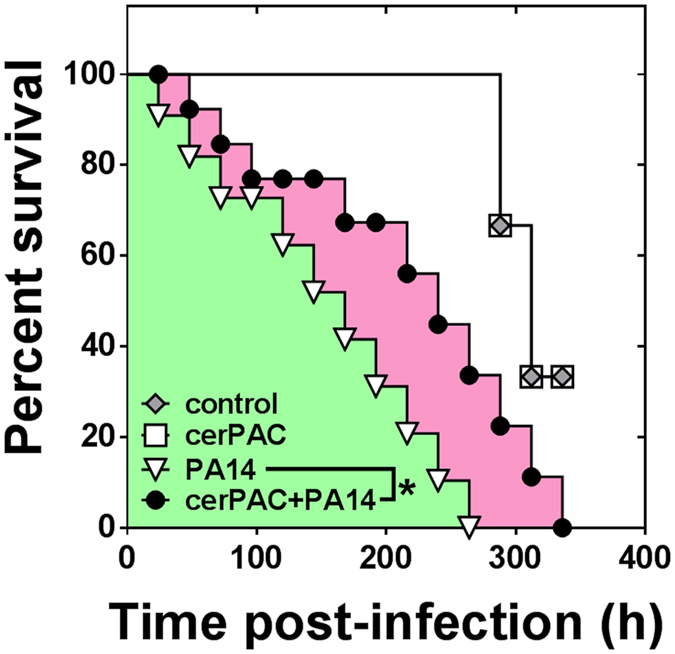
Virulence of *P. aeruginosa* PA14 towards *D. melanogaster* in absence or presence of cerPAC (200 μg mL^−1^). Mortality was scored daily for 14 days. Results represent measurements from experiments performed with triplicates, twice (*p < 0.05).

Next, to verify whether cerPAC can limit infection *in vivo*, we used a fruit fly killing assay in which we administered cerPAC to *Drosophila melanogaster* infected with WT *P. aeruginosa* PA14. As shown in Fig. 2, the median survival of *D. melanogaster* after exposure to *P. aeruginosa* was 168 h without cerPAC, but 240 h with cerPAC treatment, which is significantly (*χ*^2^ = 4.14, *df* = 1, *p* < 0.05) less virulence based on the comparison of survival curves. The survival of uninfected *D. melanogaster* was identical to the treatment with only cerPAC.

The difference in the treated or untreated PA14 strains’ ability to kill *D. melanogaster* in this feeding assay may have been due to modified survival of the bacteria on the filter papers used for exposure during incubation. To address this possibility, we analyzed the survival of PA14 on the paper discs without and with 200 μg mL^−1^ cerPAC under the same conditions as the fly feeding assay. There was no significant difference (*F*_*5,30*_ = 0.54, *p* = 0.74) in culturability of the bacterium on the filter paper discs in the absence and presence of cerPAC during incubation (see Supplementary Fig. S1), indicating that an alteration in survival ability of bacteria could not account for the observed differences in fly killing. Overall, these results indicate that cerPAC protect *D. melanogaster* from *P. aeruginosa* infection.

### Cranberry extract rich in proanthocyanidins modulates the AHL-mediated quorum sensing system in *P. aeruginosa* PA14

**Figure 3:**
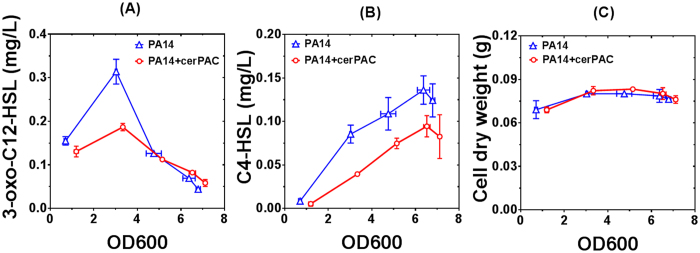
cerPAC (200 μg mL^−1^) impairs the production of AHL-type QS molecules in *P. aeruginosa* PA14. Concentrations of (**A**) 3-oxo-dodecanoyl-homoserine lactone (3-oxo-C_12_-HSL), and (**B**) butanoyl-homoserine lactone (C_4_-HSL) are shown as a function of cell growth (OD_600_). (**C**) Total cell dry weight of 3 mL culture is shown as a function of cell growth (OD_600_). Data points represent the average of triplicate experiments and the error bars show the standard deviation.

Since QS regulates multiple virulence determinants in *P. aeruginosa*, we hypothesized that cerPAC contain molecule(s) that might interfere with QS in *P. aeruginosa* PA14. Therefore, to determine the ability of cerPAC to modulate the production of the two principal AHL molecules by *P. aeruginosa* PA14, we determined AHL production kinetics in absence or presence of 200 μg mL^−1^ cerPAC. As shown in Fig, cerPAC significantly impairs the production of 3-oxo-C_12_-HSL (*t* = 7.45, *df* = 4, *p* < 0.001) and C_4_-HSL (*t* = 3.54, *df* = 4, *p* < 0.05), in *P. aeruginosa* PA14 at exponential and late stationary phase, respectively. This reduction in the production of the QS signals was observed without affecting bacterial growth (Fig. 3C).

**Figure 4:**
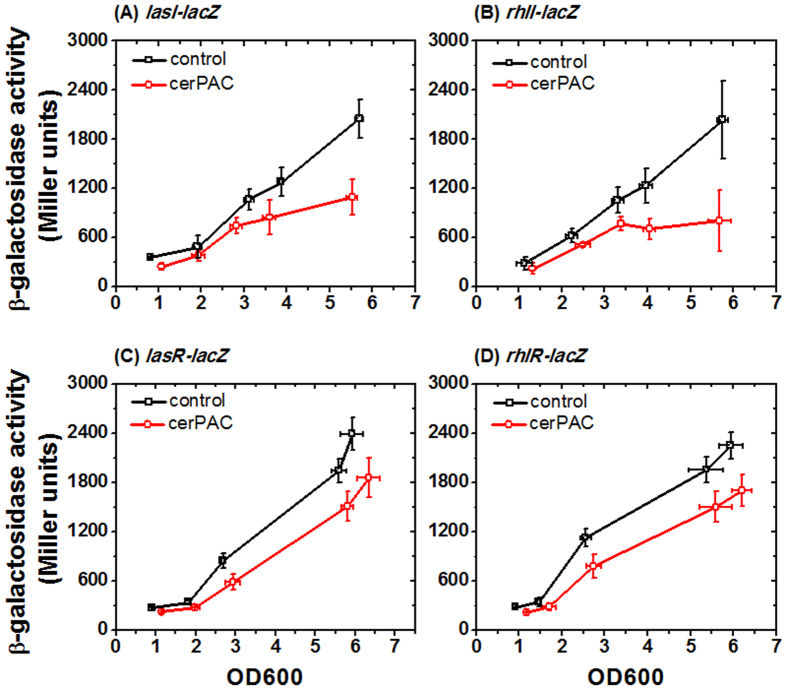
Effect of cerPAC on the expression of quorum sensing genes. *P. aeruginosa* PA14 carrying reporter fusion plasmids (**A**) *lasI*’*-lacZ* (**B**) *rhlI*’*-lacZ*, (**C**) *lasR*’*-lacZ* and (**D**) *rhlR*’*-lacZ* were grown in TSB medium without or with 200 μg mL^−1^ cerPAC, and expression was quantified by measuring β-galactosidase activity. Data points represent the average of triplicate experiments. The error bars show the standard deviation.

To understand the mechanism for the reduction in AHL levels, *β*-galactosidase transcriptional fusion reporters of *lasI* (3-oxo-C_12-_HSL synthase) and *rhlI* (C_4_-HSL synthase) were assayed in *P. aeruginosa* PA14 bioreporter strain with the same 200 μg mL^−1^ cerPAC exposure. These bioassays revealed that expression of both AHL synthase genes (*lasI* and *rhlI*) is repressed by cerPAC (Fig. 4A,B). Similarly, we also investigated whether presence of cerPAC affects the expression of the two cognate transcriptional regulator genes *lasR* and *rhlR* using *lacZ* transcriptional fusion reporters. Expression of both regulator gene fusions was partially repressed in the presence of cerPAC (Fig. 4C,D). Thus, cerPAC inhibits both AHL synthases and partially represses the LuxR-type regulator genes associated with the production of the two AHL signals in *P. aeruginosa* PA14.

### Cranberry extract rich in proanthocyanidins act as an antagonist of AHL-mediated quorum sensing in *P. aeruginosa* PA14

**Figure 5:**
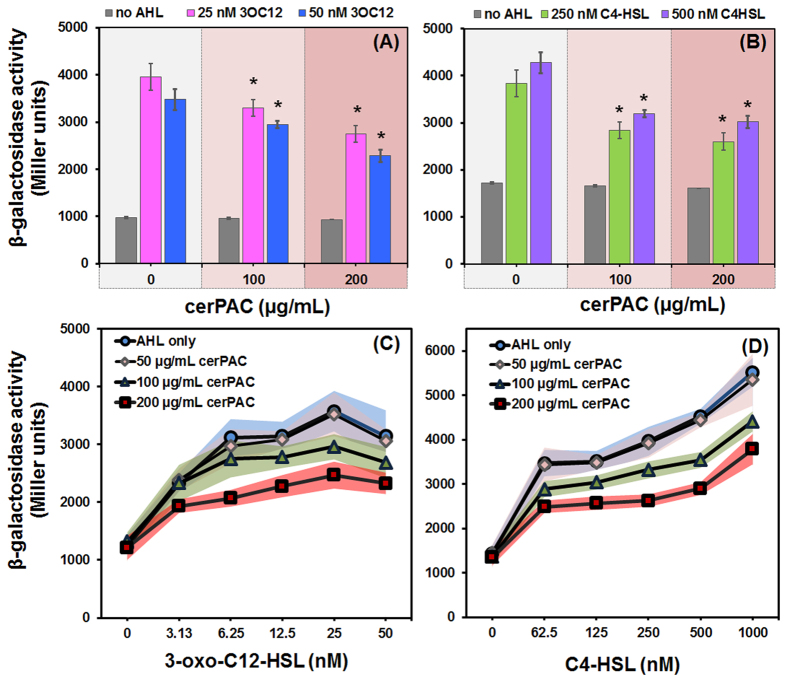
cerPAC represses AHL induction of LasR- and RhlR-controlled regulation in *P. aeruginosa* PA14. (**A**) LasR activation of *lasI-lacZ* activity in ∆*lasI*- mutant of PA14, (**B**) RhlR activation of *rhlI-lacZ* activity in ∆*rhlI*- mutant of PA14. Titration for activation of (**C**) *lasI-lacZ* in ∆*lasI*- mutant of PA14 and (**D**) *rhlI-lacZ* in ∆*rhlI*- mutant of PA14 in absence and presence of cerPAC. Error bars (**A,B**) and shaded errors (**C,D**) represent SD of triplicate assays. Statistically significant differences are indicated for each sample treated with cerPAC and each autoinducer compared to the sample treated with the corresponding concentration of each autoinducer (*p < 0.05).

Considering that AHLs act as autoinducing ligands of LasR and RhlR, we hypothesized that cerPAC component(s) interfere with LasR/RhlR activation by AHLs. We thus investigated whether cerPAC affects LasR and/or RhlR induction by exogenous AHLs using bioreporter AHL-negative PA14 mutants with *lacZ* fusions. As expected, when 3-oxo-C_12_-AHL or C_4_-HSL were supplied to their respective bioreporters, they activated the expression of *lasI* and *rhlI,* respectively (Fig. 5A,B). While cerPAC had no effect on the activity of the reporters, there was a significant (*p* < 0.05) reduced activation by either AHLs in presence of cerPAC (Fig. 5A,B). This indicates that cerPAC partially inhibits the activation of both LasR- and RhlR-directed transcription of *lasI* and *rhlI*, respectively, the primary targets of these LuxR-type regulators. Additionally, LasR and RhlR activation titration was performed in absence and presence of three different concentrations of cerPAC, which resulted in lower activation of LasR and RhlR (Fig. 5C,D). This indicates that cerPAC can reduce the activation of both regulators by their native AHLs, likely as a potential antagonist.

### Cranberry extract rich in proanthocyanidins inhibits LasR activity without binding to AHL molecules and also interact with LasI

To assess a possible physical interaction between cerPAC components and either AHL molecule, we quantified C_4_-HSL and 3-oxo-C_12_-HSL in cell-free growth medium using an ethyl acetate extraction procedure followed by LC-MS analysis. If the cerPAC binds to the AHLs, we would expect to observe a reduction in AHL concentration due to compromised extraction. As shown in Supplementary Fig. S2, there was no difference in the concentration of AHLs with or without cerPAC, demonstrating that cerPAC components do not bind to the AHLs and therefore do not inhibit QS by physical interaction.

**Figure 6:**
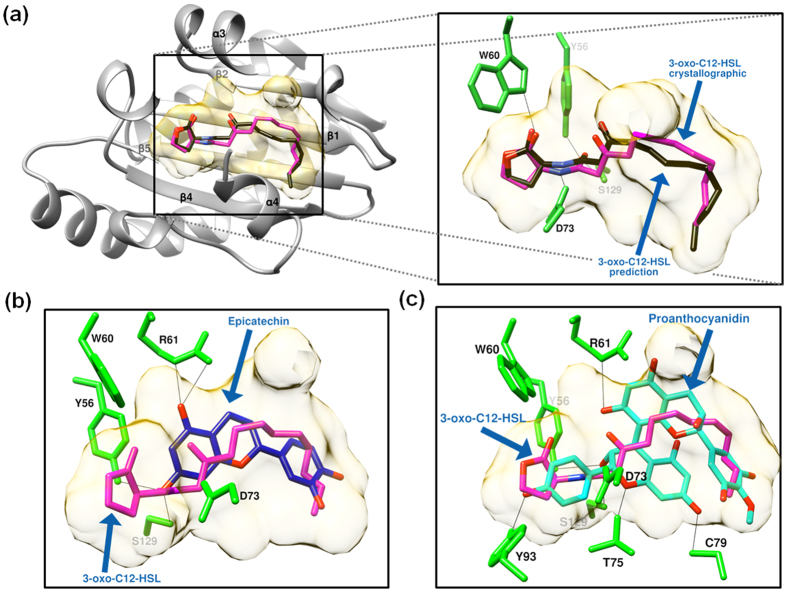
Molecular docking analysis of the LasR protein with AHL molecule and two main components of the cerPAC. (**a**) Left panel represents full view of the ribbon structure of LasR protein with the ligand binding cavity (highlighted in golden color) between four β-sheets (β1, β2, β4 and β5) and two α-helixes (α3 and α4). Upper right panel represents the inset view of docked complex with known binding position (reported crystallographic structure) of ligand 3-oxo-C12-HSL (shown in magenta color) and the predicted binding position of 3-oxo-C12-HSL (shown in black color) during *in silico* docking. The docking complexes of LasR with (**b**) the monomer of epicatechin (shown in blue color) and (**c**) the dimeric form of the epicatechin (proanthocyanidin, shown in green aqua color) are shown in the presence of 3-oxo-C12-HSL (shown in magenta color) for the comparison of binding positions. All possible hydrogen bonds are shown using black lines and binding residues shown in bright green color.

Inhibition of Las-type QS regulators’ activities by cerPAC may be due to structural interactions, important for the functional activity of transcriptional regulatory proteins. To address this possibility, *in silico* docking analysis was performed using protein structures of LasR (2UV0^46^), LasI (1RO5^47^), the monomer and dimer of epicatechin molecules (important components of cPACs^48^). To test our docking method, we compared the interaction energy scores (obtained using Moldock tools) of the predicted docking complex and the known crystallographic complex structures of the LasR with ligand 3-oxo-C_12_-HSL. The Moldock interaction energy score of −144.1 kcal mol^−1^ for the predicted complex of LasR with 3-oxo-C_12-_HSL was marginally lower than the Moldock interaction energy score of −157.5 kcal mol^−1^ obtained for the crystallographic complex of LasR with ligand 3-oxo-C_12_-HSL (Fig. 6a and see Supplementary Table S1). The epicatecin and its dimer (proanthocyanidin) molecules were docked separately in the internal cavity of LasR (Fig. 6b,c). Ligand binding domain (LBD) of LasR with a volume of 653 Å^3^, exhibits sufficient space to accommodate the monomer or dimer of epicatechin with a volume of 225 Å^3^ or 466 Å^3^, respectively. The *in silico* docking analysis suggests that the complex formation between the epicatechin and LasR, with a Moldock interaction energy score of −127.1 kcal mol^−1^, is more favorable than LasR-proanthocyanidin complex with Moldock score of −68 kcal mol^−1^ (see Supplementary Table S1). The proanthocyanidin formed six hydrogen bonds at the internal binding cavity of LasR compared to four hydrogen bonds of the LasR-3-oxo-C_12_-HSL or LasR-epicatechin complex (Fig. 6). The increase in the Moldock score for the docking complex of LasR with proanthocyanidin compared to LasR-epicatechin complex was observed due to the steric constraints of the proanthocyanidin structure in the internal cavity space of LasR identified by the comparison of their internal energies (see Supplementary Table S1).

**Figure 7:**
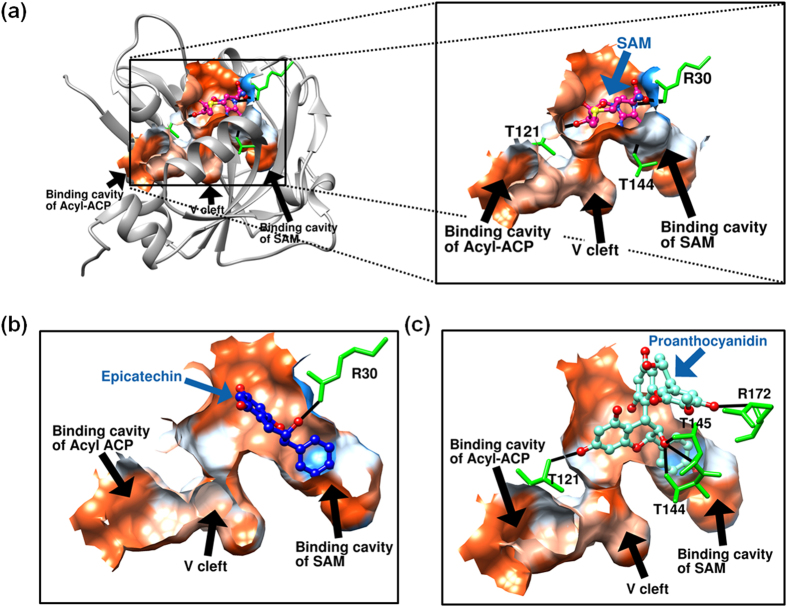
Molecular docking analysis of the LasI protein with substrate S-adenosyl L methionine (SAM) and two main components of the cerPAC. (**a**) Left panel represents full view of the ribbon structure of LasI protein with its substrates binding cavities and right panel represents the inset view of docked complex with substrate SAM (shown in magenta color). The docking complexes of LasI with (**b**) the monomer of epicatechin (shown in blue color) and (**c**) the dimeric form of the epicatechin (proanthocyanidin, shown in green aqua color) are shown with predicted binding residues (shown in bright green color). The surface structures are shown in red and blue for hydrophobic and hydrophilic attributes, respectively, and possible hydrogen bonds are depicted using black lines.

Due to the lack of crystallographic structure of LasI protein bound with its natural substrates or functional analogues, we performed *in silico* docking analysis to predict a complex of LasI with its natural substrate S-adenosyl L methionine (SAM) (Fig. 7a). This putative complex with LasI was used as a reference for both docking analyses of epicatechin and proanthocyanidin. The best five structural positions of SAM with higher interaction energies occupied the same binding cavity on the LasI protein. The docking analysis showed the formation of hydrogen bonds of SAM with residues that surround the putative binding cavity with Moldock interaction energy score of −126.2 kcal mol^−1^ (Fig. 7a and see Supplementary Table S1). The binding cavity known for the second substrate of LasI, the acyl-acyl carrier protein (acyl-ACP) was not identified as a potential binding site for either of the tested cerPAC components (epicatechin or proanthocyanidin). The LasI-epicatechin complex showed single hydrogen bond with Moldock interaction energy score of −106.8 kcal mol^−1^ (Fig. 7b and see Supplementary Table S1). The docking complex of the LasI protein with the proanthocyanidin molecule suggests the more favorable complex formation with five hydrogen bonds and Moldock interaction energy score of −153.6 kcal mol^−1^ compared to the LasI-SAM complex (Fig. 7c). This *in silico* docking analysis suggests that both main components of cerPAC have the potential to form complexes with LasR and LasI proteins to compete with their native ligands 3-oxo-C_12_-HSL and SAM, respectively.

### Cranberry extract rich in proanthocyanidins impairs AHL production in other pathogenic strains

**Figure 8:**
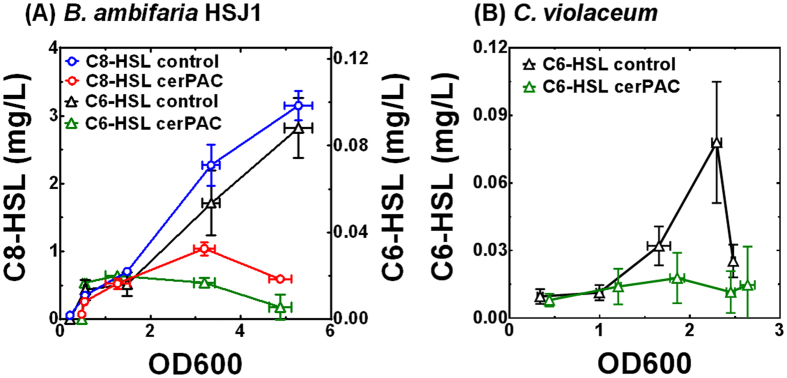
cerPAC (200 μg mL^−1^) impairs production of AHL-type QS molecules in wild type strains *B. ambifaria* HSJ1 and *C. violaceum*. Concentrations of (**A**) *N*-octanoyl-homoserine lactone (C8-HSL) and *N*-hexanoyl-homoserine lactone (C6-HSL) in *B. ambifaria* HSJ1, and (**B**) *N*-hexanoyl-homoserine lactone (C6-HSL) in *C. violaceum* are shown as a function of cell growth (OD_600_). Results are expressed as average and standard deviations (SD) from values obtained from three replications.

According to our data, cerPAC can act as a general QS inhibitor by interfering with the binding of the AHL ligand to LuxR-type transcriptional regulators. To verify that cerPAC is able to impede QS in other bacterial species, we performed an AHL production kinetics assay to examine the effect of administering cerPAC to wild type strains of *Burkholderia ambifaria* and *Chromobacterium violaceum*. The addition of cerPAC to growth medium significantly impairs the production of the two main AHLs (C_8_-HSL and C_6_-HSL) in *B. ambifaria* (Fig. 8A) and C_6_-HSL in *C. violaceum* (Fig. 8B). Since the primary target of LuxR regulators are *luxI* homologues, these observations validate the capacity of cerPAC to interfere with AHL-mediated QS in different bacterial species.

## Discussion

The identification of selective anti-virulence therapies that abolish the production of virulence determinants without affecting the viability of pathogenic bacteria would be extremely useful in combating bacterial infections caused by broad-spectrum antibiotic-resistant pathogens. The search for QS inhibitors or quenchers is a promising strategy aimed at development of innovative anti-microbial strategy to attenuate the virulence of infectious bacteria. Here, we showed that a cranberry extract enriched in PACs restricts virulence of *P. aeruginosa* in a fruit fly animal model and inhibits QS mechanisms. In addition, the cerPAC does not perturb cell viability of *P. aeruginosa*, indicating that use of these molecules may provide less selective pressure towards the development of resistance than conventional antibiotics (bactericidal and bacteriostatic, which pose strong selective pressure in any environment^45^). However, it would be naive to presume that QS inhibition is unlikely to put any selective pressures on pathogenic bacteria that may lead to resistance against QS inhibition, as quite a few studies suggest the possibility of resistance development against QS inhibition^44^,^45^. Nonetheless, it is important to continue to expand our strategies for combating pathogen resistance by identifying novel anti-microbial and anti-virulence agents^49^. Our findings have elucidated the likely mechanisms of action behind the anti-virulence efficacy of cerPAC: 1) it reduces the production of AHL signaling molecules; 2) it represses the expression of the QS regulators LasR and RhlR and autoinducer synthases LasI and RhlI; 3) it antagonizes the activation of LasR and RhlR by their cognate autoinducers; and 4) epicatechin and proanthocyanidin, the main components of cerPAC, are modeled *in silico* to interact with the LBD of LasR and LasI. In addition, cerPAC also inhibits AHL production in strains of the Gram-negative species *B. ambifaria* and *C. violaceum.* Thus, cerPAC could have anti-virulence activity against various pathogens with clinical importance.

For *in vivo* study, we used a fly feeding assay which represents a long-term infection model and involves feeding starved flies with bacterial cultures. This method is better adapted to chronic infections compared to the fly nicking model^50^. A dose of cerPAC was supplied at the start of infection and virulence was subsequently reduced, indicating that a cranberry extract enriched in PACs could function as a prophylactic. These results, when considered with other literature, indicate that the use of effective prophylactic molecules with anti-virulence activity, specifically for *P. aeruginosa*, could be a best practice in the clinical setting^51^. It is noteworthy that the extract used herein contains approximately 95% proanthocyanidins, and thus, it is presumed that the bioactivity observed can be mostly attributed to these molecules. Nonetheless, there is also a possibility that other, as yet unidentified molecules present in the cranberry extract, also act as potent quorum sensing inhibitors. Additional studies conducted with fractionated cranberry extracts are needed to achieve more insights into the bioactivity of specific fractions of cranberry derivatives.

That a cerPAC alone inhibits QS has not been previously reported in the peer-reviewed scientific literature. An earlier study reported the impairment of QS in *P. aeruginosa* in the presence of a commercial cranberry juice cocktail^52^; however, the mechanisms of action of this complex mixture of molecules was not identified. We targeted Las and Rhl QS systems because they are at the top of the *P. aeruginosa* quorum sensing hierarchy^33^. Both AHL molecules induce their own production and activate the corresponding LuxR-type transcriptional regulators LasR and RhlR^36^,^53^. In the presence of the cranberry extract, we observed impairment in AHL production, along with reduced gene expression of AHL synthase (LasI and RhlI) and partial repression of their regulators (LasR and RhlR). Similar observations have been made using other QS inhibitor molecules^51^,^54^,^55^,^56^. Interestingly, we found that cerPAC, a potent *in vivo* inhibitor, is an effective antagonist of both LasR and RhlR, two regulators that act reciprocally on key virulence determinants^33^,^51^,^57^. We propose that anti-virulence efficacy of the cerPAC is essentially due to its interference with the LasIR and RhlIR-dependent QS regulatory circuitry, though we cannot exclude a multifactorial effect.

Previous studies have identified anti-QS and anti-biofilm candidate molecules effective against *P. aeruginosa*^17^,^43^,^44^,^58^. Among them, plant-derived natural flavonoid and phenolic compounds are commonly studied for the inhibition of *P. aeruginosa* biofilm^17^,^43^ and QS^43^. The structure of a typical PAC is tetrameric in nature, composed of epicatechin units with one A-type linkage^48^. Catechin, an epimer of epicatechin, inhibits biofilm formation and QS-controlled virulence factors^59^. Consequently, other structurally-similar flavonols, such as baicalein^60^, naringenin^61^ and quercetin^55^, are also anti-QS and anti-biofilm compounds. Thus, we docked monomer and dimer (proanthocyanidin) of epicatechin molecule onto LasR and LasI proteins to assess the specific interaction. Our hypothesis was that these molecules would bind to LasR and LasI, occupying the crucial LBD, which would result in inhibition of *P. aeruginosa* QS systems. The successful molecular docking of epicatechin or proanthocyanidin with LasR suggested that the inactivation of transcriptional regulators may be the primary mechanism of action for the cerPAC as anti-virulence factors *in vivo*.

Our results show that a cerPAC protects *D. melanogaster* from *P. aeruginosa* likely through an inhibition of QS without negative effect on bacterial growth. Antagonist activity and *in silico* analysis projected the potential mechanism of action to the inhibition of AHL regulators. As hopeful as these anti-virulence strategies are, they emerge with a new set of preclinical and regulatory development challenges. It remains to be verified whether plant-derived compounds cause significant side-effects in animals or humans. However, one can also conceive of important clinical application for treatment of acute and chronic infections caused by *P. aeruginosa* and potentially other bacterial pathogens, as our preliminary data suggest cerPAC is a general inhibitor of AHL-mediated QS. Taken together, our results present a promising case for cerPACs as efficient QS inhibitors for the regulation of bacterial pathogenicity.

## Methods

### Cranberry-derived materials and bacterial strains

The cranberry extract rich in proanthocyanidins (cerPAC) was obtained from Ocean Spray Cranberries Inc. The supplier prepared the sample according to well established methods^62^ by enriching from cranberry fruit juice extract. While the exact composition is proprietary information undisclosed by the supplier, it contains approximately 95% proanthocyanidins. A dry powder of cerPAC was solubilized in deionized water and sterilized by filtration (0.22 μm PVDF membrane filter). Bacteria used in this study were *P. aeruginosa* strain PA14 (wild type)^63^ and isogenic QS mutant strains in *lasI*^64^, *rhlI*^64^, *lasR*^65^ and *rhlR*^53^ as well as wild type strains *Burkholderia ambifaria* HSJ1^66^, *Chromobacterium violaceum* ATCC 31532 and *Staphylococcus aureus* ATCC 25923. Plasmids carrying *lacZ* fusion with genes *lasI* (pSC11^67^, transcriptional fusion; pME3853^68^, translational fusion)*, lasR* (pPCS1001^36^, transcriptional fusion), *rhlI* (pMW305^69^, transcriptional fusion; pME3846^68^, translational fusion) and *rhlR* (pPCS1002^36^, transcriptional fusion) were introduced into appropriate *P. aeruginosa* PA14 QS mutant strains by electroporation, as described previously^53^. All bacterial strains were preserved in glycerol stock (15% v/v) culture at −80 °C and cultured in Tryptone Soy Broth (TSB) medium, with antibiotics if required for plasmid maintenance: tetracycline (75 mg L^−1^), carbenicillin (300 mg L^−1^), gentamicin (100 mg L^−1^), streptomycin (250 mg L^−1^) and spectinomycin (250 mg L^−1^).

### Phenotypic assay

To assess LasB elastolytic activity^53^,^70^, filter-sterilized culture supernatant samples (100 μL) from late stationary phase cultures of strain PA14 were mixed with 5 mg elastin Congo red reagent (Sigma-Aldrich) and 300 μL 0.1 M Tris-HCl (pH 7.2). Release of Congo red from degraded elastin was measured as A_495_ after 2 h of incubation at 37 °C with shaking at 100 rpm, followed by centrifugation. For assessment of LasA staphylolytic activity^53^, 5 ml of *S. aureus* ATCC 25923 overnight cultures were boiled for 15 min, and 100 μl were mixed with 300 μl of filtered culture supernatants of PA14. The OD_600_ was measured after 2 h of incubation at 37 °C and 100 rpm. To analyze alkaline protease (AprA) activity^71^, filter-sterilized culture supernatant samples (200 μL) from late stationary phase cultures of PA14 were vortexed with 25 mg of Hide–Remazol Brilliant Blue R powder (Sigma-Aldrich) in 800 μL of 20 mM Tris-HCl buffer (pH 8.0) containing 1 mM CaCl_2_. The tube was then incubated at 37 °C at 150 rpm for 1 h. The insoluble hide azure blue was removed by centrifugation at 10,000 × *g* for 4 min at 4 °C and the absorption of the supernatant was measured at 595 nm. All experiments were carried out in triplicate.

### Infection of *Drosophila melanogaster*

Fruit flies (*D. melanogaster*) were infected orally in fly feeding assay as before^50^,^72^, with some modifications. Briefly, flies were anesthetized under a gentle stream of carbon dioxide. Male flies (3- to 5-days-old) were starved of food and water for 5–6 h and separated into vials (10 per vial) containing 5 ml of 5% sucrose agar (sterile) without and with 200 μg mL^−1^ cerPAC and 2.3-cm filter paper disks (sterile) containing freshly grown bacterial culture suspension. To achieve this freshly grown culture, an overnight PA14 culture was inoculated in 6 mL TSB culture and incubated at 37 °C and 100 rpm until OD_600_ = 3.0. This culture was centrifuged at 12,000× *g* for 1 min and the resulting pellet resuspended in 150 μL of sterile 5% sucrose, without and with 200 μg mL^−1^ cerPAC. All filters were soaked appropriately with this culture suspension, along with sucrose agar, in feeding vials prior to transferring flies into the vial. Separate feeding vials soaked with 150 μL of 5% sucrose without and with 200 μg mL^−1^ cerPAC were used as negative controls for each experiment. Post-infection mortality of flies was monitored daily for 14 days, with each treatment tested twice in triplicate.

### LC-MS analyses

Specific estimation of AHL molecules was achieved by LC-MS in the positive electrospray ionization (ESI+) mode, combined with the MRM mode, as described previously^64^,^65^,^73^.

Samples of PA14 culture exposed to cerPAC were retrieved at different time points and OD_600_ was measured. An aliquot of methanolic internal standard was mixed with each sample to adjust final concentration 3 mg L^−1^ of 5,6,7,8-tetradeutero-4-hydroxy-2-heptylquinoline (HHQ-d_4_) and 6 mg L^−1^ of 5,6,7,8-tetradeutero-3,4-dihydroxy-2-heptylquinoline (PQS-d_4_)^73^. All culture samples were vortex-mixed and extracted twice with ethyl acetate (1:1, vol:vol), each ethyl acetate extract pooled and evaporated at 30 °C under a gentle stream of nitrogen. The residue was then resuspended in acidified acetonitrile (HPLC grade, containing 1% ACS grade acetic acid) at ten times the initial concentration and 20 μL aliquots were injected for LC-MS analysis^73^ (more details are in Supplementary Information).

### *β*-galactosidase assay for LacZ expression

*β-*galactosidase activity was measured as described by Miller^74^, with slight modifications as reported previously^53^. Briefly, cells were grown in TSB without and with cerPAC to various cell densities. Samples of cell culture were retrieved at different time points and diluted in Z-buffer (Na_2_HPO_4_ 0.06 M; NaH_2_PO_4_ 0.04 M; KCl 0.01 M; MgSO_4_.7H_2_O 0.001 M; β-mercaptoethanol 0.05 M; pH 7.0). Cells in Z-buffer were permeabilized by the addition of one drop of 0.1% SDS and two drops of chloroform. Then, 200 μL of 4 mg mL^−1^ ONPG was added to each reaction mixture, and enzyme reaction was stopped using 200 μL of 1 M Na_2_CO_3_. Cell debris were separated by centrifugation at 14,000× *g* for 30 sec and color development was monitored at 420 nm. β-galactosidase activity was expressed in Miller units (MU), calculated as follows: 1,000 × OD_420_/T (min) × V (mL) × OD_600_.

### Antagonists/agonists assay

To evaluate the activity of cerPAC as antagonists/agonists against the natural AHL ligand of LasR or RhlR, 3-oxo-C_12_-HSL (Sigma-Aldrich) and C_4_-HSL (Cayman Chemical) were used as inducers in this assay. The AHL-deficient strain that has been engineered to produce β-galactosidase upon activation of LasR by 3-oxo-C_12_-HSL [∆*lasI* (*lasI-lacZ*; pME3853)] and RhlR by C_4_-HSL [∆*rhlI* (*rhI-lacZ*; pME3846)], were grown overnight in TSB medium. The overnight culture was diluted in fresh TSB and was grown to achieve an OD_600_ = 0.3. An appropriate amount of sterilized cerPAC stock solution prepared in MilliQ water was added to sterile culture tube containing TSB. For control condition, either 3-oxo-C_12_-HSL or C_4_-HSL (stock solution in DMSO as a control) was added to sterile culture tube containing TSB, final DMSO concentration (after addition of cells) did not exceed 0.5% v/v. Bacterial cells were added to TSB (final OD_600_ = 0.05) without and with cerPAC approximately 30 min prior to the addition of the AHL at a final concentration of 3.13–50 nM (for 3-oxo-C_12_-HSL) or 62.5–1000 nM (for C_4_-HSL), to achieve final volume of 2 mL. Culture tubes were incubated at 37 °C for 3 h under shaking at 200 rpm, measurement of cell OD_600_ and β-galactosidase assay were performed at the regular time intervals after 3 h of incubation. The concentration of cerPAC that reduced or increased the β-galactosidase activity compared to controls containing 3-oxo-C_12_-HSL or C_4_-HSL (without cerPAC) was considered to determine antagonist or agonist activity.

### *In silico* docking analysis

To understand the interaction between components of the cerPAC with LasR and LasI protein structures, we performed virtual docking using the Piecewise Linear Potential and Lennard-Jones algorithms that can identify steric and hydrogen bonding interactions, and the Coulomb potential for electrostatic forces. *In silico* docking analysis was performed using the Molegro Virtual Docker 6.0 suite without the incorporation of water molecules. To maintain the search robustness, twenty rounds of iteration were used for each docking process. The S-adenosyl L methionine (NCBI Pubchem CID 34756) and the 3-oxo-C_12_-HSL (NCBI Pubchem CID 127864) molecular structures were used as native ligand molecules, for the LasI (RCSB Protein data base ID 1RO5^47^) and LasR (RCSB Protein data base ID 2UV0) proteins, respectively. The components of cerPAC, epicatechin (NCBI PubChem CID 182232) and proanthocyanidin (NCBI PubChem CID 108065) molecular structures were used as ligands in virtual docking for both proteins. The MolDock search tool^75^, that combines guided differential evolution and a cavity prediction algorithm was used for docking scores (in kcal mol^−1^) based on the interaction energies of each complex. The best five positions with high Moldock interaction energies were sampled and compared in every complex computed. The Computed Atlas of Surface Topography^76^ of proteins was used to explore the volumes of the cavities in the target proteins (http://sts.bioe.uic.edu/castp/index.php). The molecular graphics and analyses were performed using the UCSF Chimera version 1.1^77^.

### Statistical analysis

All experiments were repeated in quadruplicate, unless otherwise specified. Fruit fly survival curves were prepared using GraphPad Prism 6 (GraphPad Software, Inc., San Diego, CA) to perform a statistical Log-rank (Mantel–Cox) test. Data represented are the means of replicates and the differences among the control, and results were analyzed by using one way or two way ANOVA as required.

## Additional Information

**How to cite this article**: Maisuria, V. B. *et al.* Cranberry-derived proanthocyanidins impair virulence and inhibit quorum sensing of *Pseudomonas aeruginosa. Sci. Rep.* **6**, 30169; doi: 10.1038/srep30169 (2016).

## Supplementary Material

Supplementary Information

## References

[b1] Lesic B. *et al.* Inhibitors of pathogen intercellular signals as selective anti-infective compounds. PLoS Pathog. 3, 1229–1239 (2007). 17941706 10.1371/journal.ppat.0030126 PMC2323289

[b2] Shryock T. R. The future of anti-infective products in animal health. Nat Rev Micro. 2, 425–430 (2004). 10.1038/nrmicro887 15100695

[b3] Berdy J. Thoughts and facts about antibiotics: where we are now and where we are heading. J Antibiot (Tokyo) 65, 385–395 (2012). 22511224 10.1038/ja.2012.27

[b4] Jakobsen T. H., Bjarnsholt T., Jensen P. O., Givskov M. & Hoiby N. Targeting quorum sensing in *Pseudomonas aeruginosa* biofilms: current and emerging inhibitors. Future Microbiol. 8, 901–921 (2013). 23841636 10.2217/fmb.13.57

[b5] Blatherwick N. R. The specific role of foods in relation to the composition of the urine. Arch Intern Med. 14, 409–450 (1914).

[b6] Crellin J. K., Philpott J. & Bass A. L. T. A Reference Guide to Medicinal Plants (Duke University Press, 1990).

[b7] Yan X., Murphy B. T., Hammond G. B., Vinson J. A. & Neto C. C. Antioxidant activities and antitumor screening of extracts from cranberry fruit (*Vaccinium macrocarpon*). J Agric Food Chem. 50, 5844–5849 (2002). 12358448 10.1021/jf0202234

[b8] Eydelnant I. A. & Tufenkji N. Cranberry derived proanthocyanidins reduce bacterial adhesion to selected biomaterials. Langmuir. 24, 10273–10281 (2008). 18698853 10.1021/la801525d

[b9] Feldman M., Tanabe S., Howell A. & Grenier D. Cranberry proanthocyanidins inhibit the adherence properties of *Candida albicans* and cytokine secretion by oral epithelial cells. BMC Complement Altern Med. 12, 6 (2012). 22248145 10.1186/1472-6882-12-6 PMC3273432

[b10] Howell A. B., Vorsa N., Der Marderosian A. & Foo L. Y. Inhibition of the adherence of P-fimbriated *Escherichia coli* to uroepithelial-cell surfaces by proanthocyanidin extracts from cranberries. N Engl J Med. 339, 1085–1086 (1998). 9767006 10.1056/NEJM199810083391516

[b11] Tufenkji N., Rifai O. J., Harmidy K. & Eydelnant I. A. Cranberry derived proanthocyanidins can prevent pathogen invasion of kidney epithelial cells. Food Res Int. 43, 922–924 (2010).

[b12] Chan M. *et al.* Inhibition of bacterial motility and spreading via release of cranberry derived materials from silicone substrates. Colloids Surf B Biointerfaces. 110, 275–280 (2013). 23732805 10.1016/j.colsurfb.2013.03.047

[b13] Hidalgo G., Chan M. & Tufenkji N. Inhibition of *Escherichia coli* CFT073 *fliC* expression and motility by cranberry materials. Appl Environ Microbiol. 77, 6852–6857 (2011). 21821749 10.1128/AEM.05561-11 PMC3187111

[b14] McCall J., Hidalgo G., Asadishad B. & Tufenkji N. Cranberry impairs selected behaviors essential for virulence in *Proteus mirabilis* HI4320. Can J Microbiol. 59, 430–436 (2013). 23750959 10.1139/cjm-2012-0744

[b15] O’May C., Ciobanu A., Lam H. & Tufenkji N. Tannin derived materials can block swarming motility and enhance biofilm formation in *Pseudomonas aeruginosa*. Biofouling. 28, 1063–1076 (2012). 23020753 10.1080/08927014.2012.725130

[b16] O’May C. & Tufenkji N. The swarming motility of *Pseudomonas aeruginosa* is blocked by cranberry proanthocyanidins and other tannin-containing materials. Appl Environ Microbiol. 77, 3061–3067 (2011). 21378043 10.1128/AEM.02677-10 PMC3126419

[b17] Ulrey R. K., Barksdale S. M., Zhou W. & van Hoek M. L. Cranberry proanthocyanidins have anti-biofilm properties against Pseudomonas aeruginosa. BMC Complement Altern Med. 14, 499 (2014). 25511463 10.1186/1472-6882-14-499 PMC4320558

[b18] Neto C. C., Amoroso J. W. & Liberty A. M. Anticancer activities of cranberry phytochemicals: an update. Mol Nutr Food Res. 52, S18–S27 (2008). 18504707 10.1002/mnfr.200700433

[b19] Singh A. P. *et al.* Cranberry proanthocyanidins are cytotoxic to human cancer cells and sensitize platinum-resistant ovarian cancer cells to paraplatin. Phytother Res. 23, 1066–1074 (2009). 19172579 10.1002/ptr.2667 PMC2873024

[b20] Harmidy K., Tufenkji N. & Gruenheid S. Perturbation of host cell cytoskeleton by cranberry proanthocyanidins and their effect on enteric infections. PLoS One. 6, e27267 (2011). 22076143 10.1371/journal.pone.0027267 PMC3208605

[b21] Hidalgo G. *et al.* Induction of a state of iron limitation in uropathogenic *Escherichia coli* CFT073 by cranberry-derived proanthocyanidins as revealed by microarray analysis. Appl Environ Microbiol. 77, 1532–1535 (2011). 21169441 10.1128/AEM.02201-10 PMC3067223

[b22] Di Martino P. *et al.* Reduction of *Escherichia coli* adherence to uroepithelial bladder cells after consumption of cranberry juice: a double-blind randomized placebo-controlled cross-over trial. World J Urol. 24, 21–27 (2006). 16397814 10.1007/s00345-005-0045-z

[b23] Jepson R. G., Williams G. & Craig J. C. Cranberries for preventing urinary tract infections. Cochrane Database Syst Rev. 10, Cd001321 (2012). 23076891 10.1002/14651858.CD001321.pub5 PMC7027998

[b24] Lavigne J. P., Bourg G., Combescure C., Botto H. & Sotto A. *In-vitro* and *in-vivo* evidence of dose-dependent decrease of uropathogenic *Escherichia coli* virulence after consumption of commercial *Vaccinium macrocarpon* (cranberry) capsules. Clinical Microbiology and Infection. 14, 350–355 (2008). 18190583 10.1111/j.1469-0691.2007.01917.x PMC4749672

[b25] Liu Y., Black M. A., Caron L. & Camesano T. A. Role of cranberry juice on molecular-scale surface characteristics and adhesion behavior of *Escherichia coli*. Biotechnol Bioeng. 93, 297–305 (2006). 16142789 10.1002/bit.20675

[b26] Raz R., Chazan B. & Dan M. Cranberry juice and urinary tract infection. Clinical Infectious Diseases. 38, 1413–1419 (2004). 15156480 10.1086/386328

[b27] Choi E. J., Park J. B., Yoon K. D. & Bae S. K. Evaluation of the *in vitro*/*in vivo* potential of five berries (bilberry, blueberry, cranberry, elderberry, and raspberry ketones) commonly used as herbal supplements to inhibit uridine diphospho-glucuronosyltransferase. Food Chem Toxicol. 72, 13–19 (2014). 24997313 10.1016/j.fct.2014.06.020

[b28] Diarra M. S. *et al.* *In vitro* and *in vivo* antibacterial activities of cranberry press cake extracts alone or in combination with beta-lactams against *Staphylococcus aureus*. BMC Complement Altern Med. 13, 90 (2013). 23622254 10.1186/1472-6882-13-90 PMC3641957

[b29] Wu D. C., Chan W. W., Metelitsa A. I., Fiorillo L. & Lin A. N. *Pseudomonas* skin infection: clinical features, epidemiology, and management. Am J Clin Dermatol. 12, 157–169 (2011). 21469761 10.2165/11539770-000000000-00000

[b30] de Bentzmann S. & Plesiat P. The *Pseudomonas aeruginosa* opportunistic pathogen and human infections. Environ Microbiol. 13, 1655–1665 (2011). 21450006 10.1111/j.1462-2920.2011.02469.x

[b31] Navon-Venezia S., Ben-Ami R. & Carmeli Y. Update on *Pseudomonas aeruginosa* and *Acinetobacter baumannii* infections in the healthcare setting. Curr Opin Infect Dis. 18, 306–313 (2005). 15985826 10.1097/01.qco.0000171920.44809.f0

[b32] Bjarnsholt T. & Givskov M. The role of quorum sensing in the pathogenicity of the cunning aggressor *Pseudomonas aeruginosa*. Anal Bioanal Chem. 387, 409–414 (2007). 17019573 10.1007/s00216-006-0774-x

[b33] Lee J. & Zhang L. The hierarchy quorum sensing network in *Pseudomonas aeruginosa*. Protein Cell 6, 26–41 (2015). 25249263 10.1007/s13238-014-0100-x PMC4286720

[b34] Williams P. & Camara M. Quorum sensing and environmental adaptation in *Pseudomonas aeruginosa*: a tale of regulatory networks and multifunctional signal molecules. Curr Opin Microbiol. 12, 182–191 (2009). 19249239 10.1016/j.mib.2009.01.005

[b35] Latifi A., Foglino M., Tanaka K., Williams P. & Lazdunski A. A hierarchical quorum-sensing cascade in *Pseudomonas aeruginosa* links the transcriptional activators LasR and RhIR (VsmR) to expression of the stationary-phase sigma factor RpoS. Mol Microbiol. 21, 1137–1146 (1996). 8898383 10.1046/j.1365-2958.1996.00063.x

[b36] Pesci E. C., Pearson J. P., Seed P. C. & Iglewski B. H. Regulation of las and rhl quorum sensing in *Pseudomonas aeruginosa*. J Bacteriol. 179, 3127–3132 (1997). 9150205 10.1128/jb.179.10.3127-3132.1997 PMC179088

[b37] Lee J. *et al.* A cell-cell communication signal integrates quorum sensing and stress response. Nat Chem Biol. 9, 339–343 (2013). 23542643 10.1038/nchembio.1225

[b38] Hentzer M. *et al.* Inhibition of quorum sensing in *Pseudomonas aeruginosa* biofilm bacteria by a halogenated furanone compound. Microbiology. 148, 87–102 (2002). 11782502 10.1099/00221287-148-1-87

[b39] Schuster M., Lostroh C. P., Ogi T. & Greenberg E. P. Identification, timing, and signal specificity of *Pseudomonas aeruginosa* quorum-controlled genes: a transcriptome analysis. J Bacteriol. 185, 2066–2079 (2003). 12644476 10.1128/JB.185.7.2066-2079.2003 PMC151497

[b40] Tang H. B. *et al.* Contribution of specific *Pseudomonas aeruginosa* virulence factors to pathogenesis of pneumonia in a neonatal mouse model of infection. Infect Immun. 64, 37–43 (1996). 8557368 10.1128/iai.64.1.37-43.1996 PMC173724

[b41] Gonzalez J. E. & Keshavan N. D. Messing with bacterial quorum sensing. Microbiol Mol Biol Rev. 70, 859–875 (2006). 17158701 10.1128/MMBR.00002-06 PMC1698510

[b42] Scutera S., Zucca M. & Savoia D. Novel approaches for the design and discovery of quorum-sensing inhibitors. Expert Opin Drug Discov. 9, 353–366 (2014). 24597980 10.1517/17460441.2014.894974

[b43] Rasamiravaka T., Labtani Q., Duez P. & El Jaziri M. The formation of biofilms by *Pseudomonas aeruginosa*: a review of the natural and synthetic compounds interfering with control mechanisms. Biomed Res Int. 2015, 759348 (2015). 25866808 10.1155/2015/759348 PMC4383298

[b44] Garcia-Contreras R., Maeda T. & Wood T. K. Resistance to quorum-quenching compounds. Appl Environ Microbiol. 79, 6840–6846 (2013). 24014536 10.1128/AEM.02378-13 PMC3811534

[b45] Defoirdt T., Boon N. & Bossier P. Can bacteria evolve resistance to quorum sensing disruption? PLoS Pathog. 6, e1000989 (2010). 20628566 10.1371/journal.ppat.1000989 PMC2900297

[b46] Bottomley M. J., Muraglia E., Bazzo R. & Carfi A. Molecular insights into quorum sensing in the human pathogen *Pseudomonas aeruginosa* from the structure of the virulence regulator LasR bound to its autoinducer. J Biol Chem. 282, 13592–13600 (2007). 17363368 10.1074/jbc.M700556200

[b47] Gould T. A., Schweizer H. P. & Churchill M. E. Structure of the *Pseudomonas aeruginosa* acyl-homoserinelactone synthase LasI. Mol Microbiol. 53, 1135–1146 (2004). 15306017 10.1111/j.1365-2958.2004.04211.x

[b48] Neto C. C. Cranberry and its phytochemicals: a review of *in vitro* anticancer studies. J Nutr. 137, 186S–193S (2007). 17182824 10.1093/jn/137.1.186S

[b49] Njoroge J. & Sperandio V. Jamming bacterial communication: new approaches for the treatment of infectious diseases. EMBO Mol Med. 1, 201–210 (2009). 20049722 10.1002/emmm.200900032 PMC2801573

[b50] Lutter E. I., Faria M. M., Rabin H. R. & Storey D. G. *Pseudomonas aeruginosa* cystic fibrosis isolates from individual patients demonstrate a range of levels of lethality in two Drosophila melanogaster infection models. Infect Immun. 76, 1877–1888 (2008). 18285499 10.1128/IAI.01165-07 PMC2346680

[b51] O’Loughlin C. T. *et al.* A quorum-sensing inhibitor blocks *Pseudomonas aeruginosa* virulence and biofilm formation. Proc Natl Acad Sci USA 110, 17981–17986 (2013). 24143808 10.1073/pnas.1316981110 PMC3816427

[b52] Harjai K., Gupta R. K. & Sehgal H. Attenuation of quorum sensing controlled virulence of *Pseudomonas aeruginosa* by cranberry. Indian J Med Res. 139, 446–453 (2014). 24820840 PMC4069740

[b53] Dekimpe V. & Deziel E. Revisiting the quorum-sensing hierarchy in *Pseudomonas aeruginosa*: the transcriptional regulator RhlR regulates LasR-specific factors. Microbiology. 155, 712–723 (2009). 19246742 10.1099/mic.0.022764-0

[b54] Borlee B. R., Geske G. D., Blackwell H. E. & Handelsman J. Identification of synthetic inducers and inhibitors of the quorum-sensing regulator LasR in *Pseudomonas aeruginosa* by high-throughput screening. Appl Environ Microbiol. 76, 8255–8258 (2010). 20935125 10.1128/AEM.00499-10 PMC3008242

[b55] Gopu V., Meena C. K. & Shetty P. H. Quercetin Influences Quorum Sensing in Food Borne Bacteria: *In-Vitro* and In-Silico Evidence. PLoS One. 10, e0134684 (2015). 26248208 10.1371/journal.pone.0134684 PMC4527846

[b56] Rasamiravaka T. *et al.* *Pseudomonas aeruginosa* Biofilm Formation and Persistence, along with the Production of Quorum Sensing-Dependent Virulence Factors, Are Disrupted by a Triterpenoid Coumarate Ester Isolated from Dalbergia trichocarpa, a Tropical Legume. PLoS One. 10, e0132791 (2015). 26186595 10.1371/journal.pone.0132791 PMC4505864

[b57] Parsek M. R. & Greenberg E. P. Acyl-homoserine lactone quorum sensing in gram-negative bacteria: a signaling mechanism involved in associations with higher organisms. Proc Natl Acad Sci USA 97, 8789–8793 (2000). 10922036 10.1073/pnas.97.16.8789 PMC34013

[b58] Rasmussen T. B. & Givskov M. Quorum-sensing inhibitors as anti-pathogenic drugs. Int J Med Microbiol. 296, 149–161 (2006). 16503194 10.1016/j.ijmm.2006.02.005

[b59] Vandeputte O. M. *et al.* Identification of catechin as one of the flavonoids from *Combretum albiflorum* bark extract that reduces the production of quorum-sensing-controlled virulence factors in *Pseudomonas aeruginosa* PAO1. Appl Environ Microbiol. 76, 243–253 (2010). 19854927 10.1128/AEM.01059-09 PMC2798626

[b60] Zeng Z. *et al.* Virtual screening for novel quorum sensing inhibitors to eradicate biofilm formation of *Pseudomonas aeruginosa*. Appl Microbiol Biotechnol. 79, 119–126 (2008). 18330563 10.1007/s00253-008-1406-5

[b61] Vandeputte O. M. *et al.* The flavanone naringenin reduces the production of quorum sensing-controlled virulence factors in *Pseudomonas aeruginosa* PAO1. Microbiology. 157, 2120–2132 (2011). 21546585 10.1099/mic.0.049338-0

[b62] Howell A. B. *et al.* A-type cranberry proanthocyanidins and uropathogenic bacterial anti-adhesion activity. Phytochemistry. 66, 2281–2291 (2005). 16055161 10.1016/j.phytochem.2005.05.022

[b63] Rahme L. G. *et al.* Common virulence factors for bacterial pathogenicity in plants and animals. Science. 268, 1899–1902 (1995). 7604262 10.1126/science.7604262

[b64] Deziel E. *et al.* The contribution of MvfR to *Pseudomonas aeruginosa* pathogenesis and quorum sensing circuitry regulation: multiple quorum sensing-regulated genes are modulated without affecting lasRI, rhlRI or the production of N-acyl-L-homoserine lactones. Mol Microbiol. 55, 998–1014 (2005). 15686549 10.1111/j.1365-2958.2004.04448.x

[b65] Deziel E. *et al.* Analysis of *Pseudomonas aeruginosa* 4-hydroxy-2-alkylquinolines (HAQs) reveals a role for 4-hydroxy-2-heptylquinoline in cell-to-cell communication. Proc Natl Acad Sci USA 101, 1339–1344 (2004). 14739337 10.1073/pnas.0307694100 PMC337054

[b66] Vial L. *et al.* *Burkholderia pseudomallei, B. thailandensis,* and *B. ambifaria* produce 4-hydroxy-2-alkylquinoline analogues with a methyl group at the 3 position that is required for quorum-sensing regulation. J Bacteriol. 190, 5339–5352 (2008). 18539738 10.1128/JB.00400-08 PMC2493281

[b67] Chugani S. A. *et al.* QscR, a modulator of quorum-sensing signal synthesis and virulence in *Pseudomonas aeruginosa*. Proc Natl Acad Sci USA 98, 2752–2757 (2001). 11226312 10.1073/pnas.051624298 PMC30211

[b68] Pessi G. *et al.* The global posttranscriptional regulator RsmA modulates production of virulence determinants and N-acylhomoserine lactones in *Pseudomonas aeruginosa*. J Bacteriol. 183, 6676–6683 (2001). 11673439 10.1128/JB.183.22.6676-6683.2001 PMC95500

[b69] Whiteley M., Parsek M. R. & Greenberg E. P. Regulation of quorum sensing by RpoS in *Pseudomonas aeruginosa*. J Bacteriol. 182, 4356–4360 (2000). 10894749 10.1128/jb.182.15.4356-4360.2000 PMC101961

[b70] Bjorn M. J., Sokol P. A. & Iglewski B. H. Influence of iron on yields of extracellular products in *Pseudomonas aeruginosa* cultures. J Bacteriol. 138, 193–200 (1979). 108250 10.1128/jb.138.1.193-200.1979 PMC218257

[b71] Howe T. R. & Iglewski B. H. Isolation and characterization of alkaline protease-deficient mutants of *Pseudomonas aeruginosa in vitro* and in a mouse eye model. Infect Immun. 43, 1058–1063 (1984). 6421735 10.1128/iai.43.3.1058-1063.1984 PMC264293

[b72] Apidianakis Y. & Rahme L. G. *Drosophila melanogaster* as a model host for studying *Pseudomonas aeruginosa* infection. Nat Protoc. 4, 1285–1294 (2009). 19680242 10.1038/nprot.2009.124

[b73] Lepine F. & Deziel E. Liquid chromatography/mass spectrometry for the detection and quantification of N-acyl-L-homoserine lactones and 4-hydroxy-2-alkylquinolines. Methods Mol Biol. 692, 61–69 (2011). 21031304 10.1007/978-1-60761-971-0_5

[b74] Miller F. Glycopeptides of human immunoglobulins. 3. The use and preparation of specific glycosidases. Immunochemistry. 9, 217–228 (1972). 4338321

[b75] Thomsen R. & Christensen M. H. MolDock: a new technique for high-accuracy molecular docking. J Med Chem. 49, 3315–3321 (2006). 16722650 10.1021/jm051197e

[b76] Liang J., Edelsbrunner H. & Woodward C. Anatomy of protein pockets and cavities: measurement of binding site geometry and implications for ligand design. Protein Sci. 7, 1884–1897 (1998). 9761470 10.1002/pro.5560070905 PMC2144175

[b77] Pettersen E. F. *et al.* UCSF Chimera–a visualization system for exploratory research and analysis. J Comput Chem. 25, 1605–1612 (2004). 15264254 10.1002/jcc.20084

