# Supplementary material for: Cranberry-derived proanthocyanidins impair virulence and inhibit quorum sensing of Pseudomonas aeruginosa
Source: Sci Rep. 2016 Aug 9;6:30169. doi: 10.1038/srep30169 (PMC4977528; doi:10.1038/srep30169)
Supplement: Supplementary Information [file srep30169-s1.pdf]

<u>**Supplementary Information:**</u>

Cranberry-derived proanthocyanidins impair virulence and inhibit quorum sensing of *Pseudomonas aeruginosa*

Vimal B. Maisuria[1], Yossef Lopez-de Los Santos[2], Nathalie Tufenkji[1]*, Eric Déziel[2]*

[1]Department of Chemical Engineering, McGill University, 3610 University Street, Montreal, Quebec, Canada.

[2]INRS-Institut Armand-Frappier, 531 boul. des Prairies, Laval, Québec, Canada.

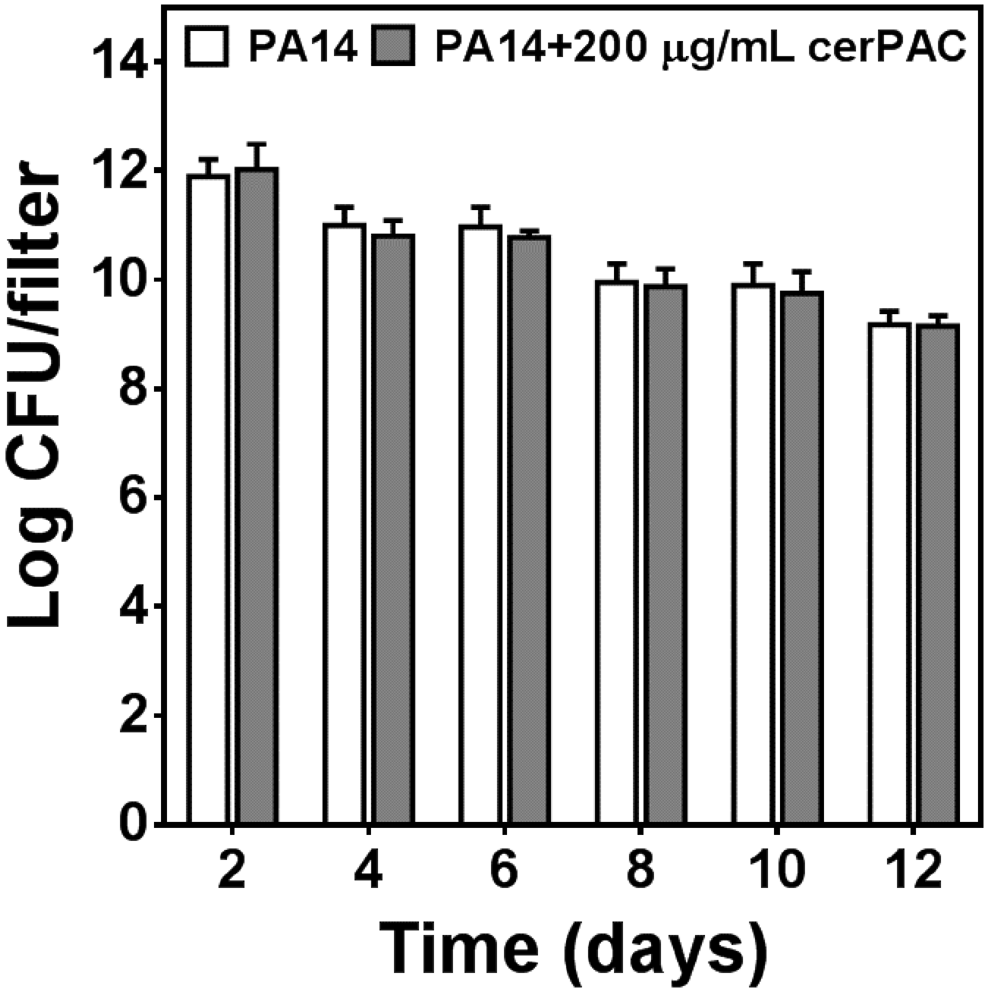

Figure S1. *S*urvival of *P. aeruginosa* PA14 on sucrose filters during the fly feeding assay. Total viable bacterial counts per filter were determined every second day for filters containing bacterial cells on sucrose agar. Average data represent data from triplicate assays and error bars represent S.D.

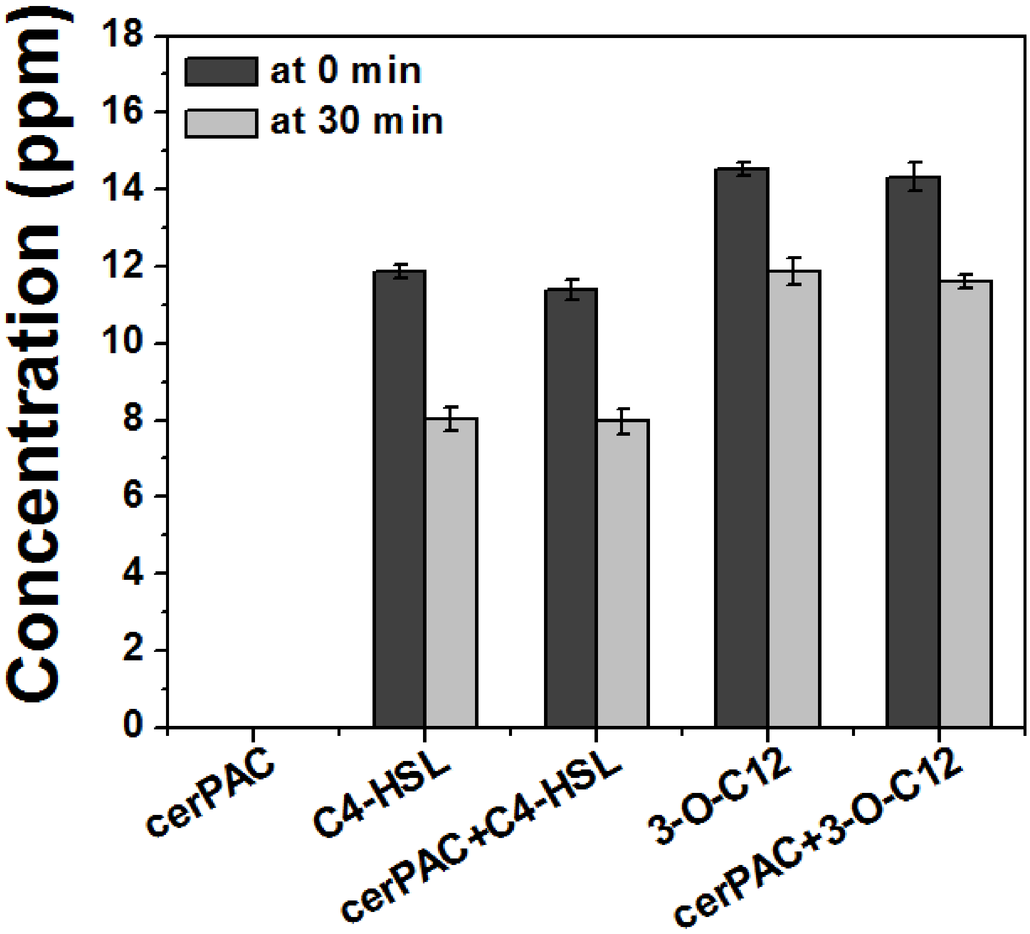

Figure S2. Interaction of cerPAC with AHL molecules in TSB medium. 15 ppm C_4-HSL and 15 ppm 3-oxo-C_{12}-HSL (3-O-C12) were added individually in sterile TSB with or without 200 µg mL^{-1} cerPAC, and concentrations were quantified by LC/MS. C_4-HSL and 3-oxo-C_{12}-HSL indicates only AHL controls. Data points are the average with standard deviation of triplicate assays.

Table S1. Interaction energies of ligand-protein complexes during *in silico* docking analysis for Las regulatory proteins.

	Interaction energy scores (kcal mol^{-1})[d] for proteins							
	LasR[c]				LasI[c]			
Ligands	E_{intra}	TS	E_{inter}	Moldock	E_{intra}	TS	E_{inter}	Moldock
3-oxo-C12-HSL(C)[a]	-8	10.4	-160.4	-157.5	NA	NA	NA	NA
3-oxo-C12-HSL(P)[b]	-12	24.3	-156.6	-144.1	NA	NA	NA	NA
S-adenosyl L methionine	NA	NA	NA	NA	-29.7	7	-103.6	-126.2
Epichatechin	14.7	0.2	-142	-127.1	12.1	0.2	-118.8	-106.8
Proanthocyanidin	80.5	2	-150.3	-68	15.6	1.3	-170.5	-153.6

[a]C, structural pose during crystallographic analysis reported by Bottomley et al. *J Biol Chem.* 282, 13592-13600 (2007)
[b]P, predicted structural pose during our docking analysis
[c]E_{intra}, internal ligand energy between atoms of the ligand (electrostatic, steric and hydrogen interactions); E_{inter}, ligand-protein interaction energy; TS, energy penalties for the internal torsional strain of a ligand; Moldock, total interaction energy using Moldock scoring function.
[d]NA, not available

Methods:

Growth kinetics

To assess growth kinetics, *P. aeruginosa* PA14 was grown in the absence or presence of cerPAC at 6.25, 25, 100, 200 and 300 µg mL^{-1}. An overnight culture of PA14, grown at 37°C with shaking at 200 rpm, was diluted 1,000-fold with TSB medium. This cell suspension containing approximately 10^6 cells mL^{-1} was aliquoted into sterile 100-well honeycomb microplates containing different amount of cerPAC and incubated at 37°C until stationary phase was reached. The OD_{600} was recorded at 30 minutes time intervals using a BioScreen C system (Growth Curves USA, Piscataway, NJ). Each condition was set-up in four replicates. The optimum concentration of cerPAC that did not hinder growth of PA14 in TSB was selected in all subsequent assays, unless otherwise noted. Similarly, another set of experiments was conducted with larger volume (3 mL) to analyze the effect of cerPAC on growth and AHL production. Samples were collected at different time points for OD_{600} and LC/MS analyses. Dry weight of the bacterial suspensions at each time point was determined using pre-weighed aluminum cups that were incubated at 65 °C for 4 h to allow water evaporation. Cups were weighed again to determine total dry weight.

Bacterial cell enumeration on filters during the fly feeding assay

Enumeration of viable bacteria on filter during the infection period was completed using separate test vials inoculated with PA14 and uninoculated control sucrose vials that were sampled on alternative days, up to 12 days during infection period. Briefly, filters from the test vials were removed in sterile environment, placed in 50 mL polypropylene tube containing 5 ml of LB broth and vortexed for 30 sec. This LB medium containing the sampled bacterial cells was serially diluted in phosphate buffer saline solution (pH 7), and 30 µL was plated onto TSB agar

and *Pseudomonas* Isolation agar (Thermo Scientific Remel, Fisher Scientific, Canada). Colonies were enumerated after incubation at 37 °C for 24 h.

Physical interaction assay

To assess any physical interaction between cerPAC and 3-oxo-C_{12}-HSL or C_4-HSL (in absence of cells), 200 µg mL^{-1} of cerPAC (dissolved in MilliQ water) was mixed individually with 15 µg mL^{-1} 3-oxo-C_{12}-HSL and 15 µg mL^{-1} C_4-HSL in TSB medium (final volume 5 mL), and incubated at 37°C for 30 min and 200 rpm (only 3-oxo-C_{12}-HSL and C_4-HSL in TSB were kept as controls). Internal standards (3 mg L^{-1} HHQ-d_4, 6 mg L^{-1} PQS-d_4, 1.3 mg L^{-1} DHQ-d_4 and 2 mg L^{-1}AA-d_4) were also added to each tube for the quantification of each AHL molecule as described previously [3]. Samples were collected before and after incubation and centrifuged at 13,000 × *g* for 15 min. Supernatants were filtered using 0.22 µm PVDF membrane filters and AHLs were extracted using ethyl acetate, followed by evaporation under a nitrogen gas stream. Concentration was analyzed using LC-MS analyses [1-3].

LC-MS analyses

The LC-MS analyses were performed with a Quattro II (Waters) triple quadrupole mass spectrometer (MS) equipped with a Z-spray interface as described previously [1-3]. Nitrogen was used for drying and argon was used as collision gas in multiple reactions monitoring (MRM) mode. HPLC (1100 HP) was equipped with a 4.6×150mm Eclipse XDB C8 column (Agilent) and the MS was connected to the HPLC through a T splitter (Valco). The third output of the splitter was fitted with a tube of internal diameter and length such that only 10% of the initial flow goes to the electrospray probe. Solvent A: ultrapure water containing 1% ACS grade acetic acid. Solvent B: acetonitrile (HPLC grade), containing 1% ACS grade acetic acid. The solvent

gradient for the chromatographic runs was as follows: from 0 to 1 min 70% solvent A; from 1 to 13 min 100% solvent B; from 13 to 23 min 100% solvent B; from 23 to 25 min 70% solvent A; from 25 to 28 min 70% solvent A. Flow rate was set at 400 µL min^{-1} split to 40 µL min^{-1} by the T splitter. The MS parameters were: positive mode; needle voltage 3.0 kV; cone 30 V; block temperature 120 °C and drying gas 150 °C; nebulising gas 20 µL min^{-1} and drying gas 200 µl min^{-1}. In full scan mode, the scanning range was set to *m*/*z* 100–400.

References

1 Déziel, E. *et al.* Analysis of *Pseudomonas aeruginosa* 4-hydroxy-2-alkylquinolines (HAQs) reveals a role for 4-hydroxy-2-heptylquinoline in cell-to-cell communication. *Proc Natl Acad Sci U S A*. **101**, 1339-1344 (2004).

2 Déziel, E. *et al.* The contribution of MvfR to *Pseudomonas aeruginosa* pathogenesis and quorum sensing circuitry regulation: multiple quorum sensing-regulated genes are modulated without affecting lasRI, rhlRI or the production of N-acyl-L-homoserine lactones. *Mol Microbiol*. **55**, 998-1014 (2005).

3 Lépine, F. & Déziel, E. Liquid chromatography/mass spectrometry for the detection and quantification of N-acyl-L-homoserine lactones and 4-hydroxy-2-alkylquinolines. *Methods Mol Biol*. **692**, 61-69 (2011).
